# COVID-19 Booster Vaccine Equity for Patients With Cancer

**DOI:** 10.1016/j.adro.2022.100939

**Published:** 2022-03-09

**Authors:** Rahul N. Prasad, Manali Patel, Joshua D. Palmer

**Affiliations:** a Department of Radiation Oncology at the Arthur G. James Cancer Hospital and Richard J. Solove Research Institute, The Ohio State University Comprehensive Cancer Center, Columbus, Ohio; b Stanford University School of Medicine and VA Palo Alto Health Care System, Stanford, California

## Abstract

COVID-19 has caused greater than 300 million documented infections worldwide including over 5 million confirmed deaths. Patients with cancer are particularly vulnerable due to a combination of disease and therapy-related effects. Available vaccines were highly effective against the original viral strains in clinical trials. However, initial vaccination efforts in this vulnerable population were impacted by federal policy that created substantial vaccine scarcity and allocation difficulties by recommending prioritization of unmanageably large patient populations including the entire elderly population and patients over the age of 16 with broadly defined, high-risk medical conditions (including cancer). We found that these overly broad recommendations led nearly two-thirds of states to elect not to give adequate vaccination priority to patients with cancer, exposing this vulnerable population to potentially preventable infection. With the virulent omicron variant spreading rapidly, there is newfound concern about waning immunity, particularly in immunocompromised populations. To address this issue, the Centers for Disease Control is recommending boosters for patients who meet age, occupational exposure, or medical criteria, in similar fashion to recommendations during the initial vaccination phase. Thus, this approach raises the question of whether state-level decisions on how to sub prioritize may inadvertently once again result in delayed immunizations for particularly vulnerable subgroups – such as patients with cancer. We discuss the implications of this public health policy on the likelihood of timely re-vaccination of patients with cancer. With the omicron variant continuing its unchecked global spread, equitable distribution of booster immunizations is critical to minimizing inherent medical and socioeconomic inequities in COVID-related morbidity and mortality.

Severe acute respiratory syndrome coronavirus 2 infection is responsible for over 300 million cases and 5 million deaths from COVID-19 worldwide.^1^ Despite the availability of highly effective vaccines,^2^^,^^3^ case counts in the United States (US) are at an all-time high at least partially due to the emergence of multiple variants that have eroded the efficacy of the initial vaccination sequence.^4^ As a result, the Centers for Disease Control and Prevention (CDC) now recommends boosters for all adults more than 6 months out from their initial vaccinations. Patients with cancer are particularly vulnerable to COVID due to both cancer-associated and immunosuppressive therapy-related effects.^5^ A systematic review of outcomes in patients with cancer showed an increased risk of COVID-related mortality,^5^ and the CDC appropriately included cancer as a high-risk condition.^6^

Because every adult in the United States is currently eligible for a booster 6 months after completion of their initial vaccination series, limited access to currently available boosters, as well as future boosters targeted at variants of concern (VOC), for patients with cancer in the US is a possibility, particularly in the context of past access issues. The CDC Advisory Committee on Immunization Practices published an overly broad prioritization scheme during initial vaccination efforts^7^ that created significant vaccine scarcity and allocation difficulties. Although health care providers and residents of long-term care facilities were included in phase 1A and phase 1B included other patients over 75, phase 1C prioritized very large patient populations such as the entire elderly population and all patients over the age of 16 with common high-risk conditions such as obesity, cancer, and type 2 diabetes.^6^^,^^7^ Eighty-one million unique people aged 16 to 64 met the initial definitions of high-risk medical conditions alone.^7^ Faced with an overwhelming number of patients meeting criteria for expedited vaccination, states had to decide how best to triage even among priority groups. The patient prioritization decisions made at the state level during initial vaccination phases have been poorly studied.

The proportion of states that included all patients over 16 years of age with a cancer diagnosis (vaccine-eligible age) in the same vaccination tier as patients over 65 and remaining essential workers during the initial phases of vaccination has not been well characterized. Through Google-based Internet searches using the terms “COVID vaccine phases,” “cancer,” and “high-risk medical conditions,” we identified every state's COVID vaccination webpage in February of 2021. Although age-based vaccine eligibility criteria were readily identifiable for each state, definitions for high-risk medical conditions including cancer were difficult to find, and identifying information regarding the vaccine eligibility for patients with cancer was challenging. Finding this information routinely required navigation through multiple webpage subdomains. These issues were likely significant barriers to information acquisition for patients. We found that 17 states gave all patients with cancer and patients over 65 the same initial immunization priority; however, nearly two-thirds opted not to follow the Advisory Committee on Immunization Practices recommendations. These 17 states included the states with the highest and lowest per capita cancer prevalence in the Unite States. The median per capita cancer prevalence for these 17 states was similar to the median for the entire country, suggesting minimal relationship between relative cancer burden and decisions on whether to prioritize vaccination of patients with cancer. Another 7 (17%) states nominally included patients with cancer in the same vaccination subtier as patients over 65, but prioritized patients over 65 by first opening vaccination slots for elderly patients. Of the 43 states that used cancer as criteria for early vaccination, 35 explicitly mentioned cancer and other high-risk conditions on their website (Fig 1). Another 8 simply directed readers to the CDC website listing high-risk conditions. One state specified its own criteria for high-risk disease that omitted cancer, and 6 states (12%) did not clearly designate whether a cancer diagnosis had any influence on vaccination eligibility.

**Fig. 1:**
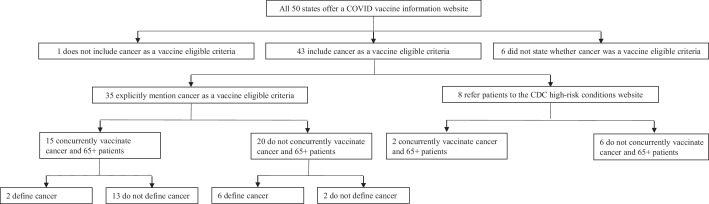
Schematic demonstrating online availability of COVID-19 vaccine eligibility information for patients with cancer at the state level during the initial phase of vaccination.

*Abbreviations:* CDC = Centers for Disease Control and Prevention.

Additionally, less than one-fifth of states (8) provided a precise definition for a cancer diagnosis meeting the criteria for vaccination. When provided, definitions for a cancer diagnosis were heterogeneous. Six of these definitions only included patients receiving active or recent treatment, 1 limited eligibility to solid tumors diagnosed within the last year or 5 years for hematologic malignancies, and 1 state included all known patients with cancer. For the remainder of states, it was unclear whether any prior cancer diagnosis was sufficient for vaccination or if current or recent treatment was necessary. This ambiguity is a potential source of confusion for patients with many highly prevalent conditions such as remote histories of breast, prostate, or cancers in remission; “preinvasive” conditions such as ductal carcinoma in situ; or “benign” tumors such as meningioma. Thus, this general lack of specific definitions for included medical conditions during initial vaccination phases was a significant source of uncertainty. Although ensuring equitable vaccine allocation is integral to combatting future VOC, benefits are minimized if patients are not aware of or are confused about their eligibility.

Despite the association between a cancer diagnosis and inferior COVID-related outcomes^5^ leading to federal guidance to include patients with cancer in the same tier as patients aged 65 to 74,^7^ we noted that nearly two-thirds of states did not give recommended prioritization to patients with cancer during the initial phase of vaccination. This discrepancy perhaps stemmed from a desire to streamline vaccination efforts to avoid waste, particularly initially at mass vaccination sites where providers were unfamiliar with patients. This decision resulted in improved access for elderly patients, including those without the tech savvy to compete online for hotly contested vaccination slots. This approach may have also been a response to the broadness of the definition of high-risk medical conditions. Patients living with a past or present cancer diagnosis are a large, heterogeneous population (over 17 million in the United States)^5^ but are just a fraction of the 81 million unique, medically high-risk patients,^7^ a category that includes even common conditions such as obesity. Vague definitions for common conditions such as body mass index > 30 led to an overwhelmingly large number of eligible patients, which likely encouraged states to de-emphasize the entire high-risk category to avoid crowding out elderly patients.^8^

Notably, although population-based studies suggest that all hospitalized, obese patients have an elevated risk of COVID-related death (odds ratio [OR], 1.28),^9^ the magnitude of the effect in young, hospitalized, COVID-positive patients appears to be greatest for the morbidly obese (OR, 2.30 compared with no obesity).^10^ Another study found that while advanced age (OR, 11.15; ≥80 vs <40) may be the greatest risk factor for death in COVID-positive patients requiring intensive care, the magnitude of the effect for active cancer (OR, 2.15) may be greater even than that for other common, high-risk conditions such as morbid obesity (OR, 1.51) or coronary artery disease (OR, 1.49).^11^ Acute troponin elevations in admitted patients have been more strongly linked to adverse COVID-related outcomes than underlying cardiac comorbidities such congestive heart failure, perhaps reflecting the prognostic importance of myocardial damage and inflammation.^12^

When it came time for boosters, initially it appeared that the CDC may have refined its prioritization strategy. In response to concerns about waning immunity^4^ and the rapidly spreading Delta variant, the CDC first recommended boosters at least 6 to 8 months after completion of the initial vaccination series for just a relatively small population of patients who were heavily immunocompromised, which included some patients with cancer receiving active therapy. However, this announcement occurred before many immunosuppressed patients exited the recommended waiting period between initial vaccination and booster eligibility, and within weeks, booster immunizations were recommended to a much broader population. This group meeting expanded booster eligibility was defined by various factors including age (≥65), exposures (health care worker or resident of long-term care facility), or a combination of age (≥50) with medical comorbidities.^13^ Roughly approximating the scale of the original priority groupings, the broadness of this new stratification once again raised the question of whether state-level decisions would inadvertently result in delayed booster immunizations for particularly vulnerable subgroups – such as patients with cancer receiving active therapy. Eventually, the emergence of the Omicron variant became an additional obstacle to booster prioritization, as it prompted the CDC to recommend boosters for *all* adults more than 6 months out from their initial vaccinations.

Of course, although vaccine scarcity was of primary concern during the initial rollout, vaccine hesitancy may have later become a bigger obstacle to booster efforts. However, hesitancy appears to be less prevalent in oncology populations. Several recent cross-sectional survey studies found that over 90% of patients with cancer were willing or would be willing to take the vaccine if recommended by their physician.^14^^,^^15^ Unfortunately, vaccine scarcity may re-emerge in the future, as the reduced efficacy of the current vaccine formulations against the Omicron variant (even relative to Delta) suggests that there is a reasonable chance that reformulated boosters specifically targeting a single (or multiple) VOC may soon be required. At first release, manufacturing and distribution may be insufficient to meet demand. If future booster rollouts continue to largely occur in existing clinics, offices, and pharmacies rather than mass vaccination centers, the primary advantage of age-based vaccination criteria, ease and rapidity of deployment, may be negated. One potential advantage of using existing health care providers with greater familiarity with patients would be the ability to use more nuanced immunization criteria that do not risk delaying vaccination for patients whose vulnerability is not captured by age alone,^8^^,^^16^ such as a young patient with cancer or one on dialysis requiring frequent therapy outside the home.

Moving forward in this pandemic, if newly formatted boosters are indeed needed as suggested by several vaccine manufacturers, more narrowly defining vague conditions meeting criteria could target efforts toward the highest risk patient subsets. For example, narrowing the definition of a cancer diagnosis meeting high-risk criteria could ensure that at least the highest risk patients with cancer receive priority vaccination. Some patients with cancer, such as long-term survivors in remission, may have a COVID-19 risk approximating a cancer-free patient of the same age. A subset of patients with immunosuppression from active therapy (such as bone marrow transplant, chemotherapy,^17^ and radiation therapy with large fields) or those with hematologic or metastatic diagnoses may predominately drive adverse outcomes.^5^ Patients with new diagnoses or active therapy could be prioritized. Of course, any stratification sufficiently broad for easy applicability risks introducing novel inequities. For example, differentiating between long-term survivors and recently diagnosed patients, as 7 states chose to do during initial vaccination efforts, risks exacerbating pre-existing health care access issues by prioritizing populations with the ability to receive regular cancer screening. Additionally, patients with screening-related malignancies for which active surveillance may be a reasonable option (such as favorable prostate cancers) may be at lower risk for severe COVID than some previously treated patients in remission. An alternative approach would be to prioritize vaccination of patients with multiple comorbidities, but this approach risks under-coverage of patients with a single severe condition. Another relatively unexplored approach is to stratify immunocompromised patients by the presence of additional comorbidities; for example, a population-study found that hospitalized patients with COVID with cancer who were smokers were at higher risk for in-hospital death then nonsmokers, and noted a trend toward increased mortality with underlying cardiovascular disease.^17^ Patients with demographic features associated with increased COVID severity, such as lower socioeconomic status or vulnerable race/ethnicity, could also be prioritized.^18^

In summary, CDC recommendations for booster vaccinations roughly mimicked guidelines during initial vaccine phases by prioritizing extremely large patient populations for expedited booster vaccinations. Based on our findings that nearly two-thirds of states elected not to give adequate vaccination prioritization to patients with cancer during the initial phases under similar guidance, if boosters targeted at VOC are eventually needed, this approach raises the question of whether state-level decisions on how to subprioritize patients may again inadvertently result in delayed immunizations for particularly vulnerable subgroups such as patients with cancer. Although boosters are not scarce at this time, reformulated boosters targeting VOC may be scarce at release, triggering supply and demand mismatches reminiscent of the initial vaccination rollout. Potential solutions include applying more targeted prioritization schemes, which, after prioritizing elderly patients (such as over 65), focus on only the highest-priority patients with high-risk medical conditions; stratifying patients by the presence of additional comorbidities; or considering high-risk demographic features. Additional opportunities for improvement include decreasing confusion through clearer definitions for conditions meeting criteria for vaccination and streamlining websites to lessen barriers to information acquisition. With the Omicron variant continuing its unchecked global spread and natural and vaccine-related immunity potentially waning, equitable distribution of booster immunizations is essential to minimizing inherent medical, age-related, and socioeconomic inequities in COVID-related morbidity and mortality between populations.
